# Use of Host Feeding Behavior and Gut Microbiome Data in Estimating Variance Components and Predicting Growth and Body Composition Traits in Swine

**DOI:** 10.3390/genes13050767

**Published:** 2022-04-26

**Authors:** Yuqing He, Francesco Tiezzi, Jicai Jiang, Jeremy T. Howard, Yijian Huang, Kent Gray, Jung-Woo Choi, Christian Maltecca

**Affiliations:** 1 Department of Animal Science, North Carolina State University, 120 W Broughton Dr, Raleigh, NC 27607, USA; jicai_jiang@ncsu.edu (J.J.); cmaltec@ncsu.edu (C.M.); 2 Department of Agriculture, Food, Environment and Forestry (DAGRI), University of Florence, Piazzale delle Cascine 18, 50144 Firenze, Italy; 3 Smithfield Premium Genetics, Rose Hill, NC 28458, USA; jhoward@smithfield.com (J.T.H.); yhuang@smithfield.com (Y.H.); kgray@smithfield.com (K.G.); 4 College of Animal Life Sciences, Division of Animal Resource Science, 1 Gangwondaehak-gil, Chuncheon-si 24341, Gangwon-do, Korea; jungwoo.kor@gmail.com

**Keywords:** feeding behavior, gut microbiome composition, prediction, growth, breeds, swine

## Abstract

The purpose of this study was to investigate the use of feeding behavior in conjunction with gut microbiome sampled at two growth stages in predicting growth and body composition traits of finishing pigs. Six hundred and fifty-one purebred boars of three breeds: Duroc (DR), Landrace (LR), and Large White (LW), were studied. Feeding activities were recorded individually from 99 to 163 days of age. The 16S rRNA gene sequences were obtained from each pig at 123 ± 4 and 158 ± 4 days of age. When pigs reached market weight, body weight (BW), ultrasound backfat thickness (BF), ultrasound loin depth (LD), and ultrasound intramuscular fat (IMF) content were measured on live animals. Three models including feeding behavior (Model_FB), gut microbiota (Model_M), or both (Model_FB_M) as predictors, were investigated. Prediction accuracies were evaluated through cross-validation across genetic backgrounds using the leave-one-breed-out strategy and across rearing environments using the leave-one-room-out approach. The proportions of phenotypic variance of growth and body composition traits explained by feeding behavior ranged from 0.02 to 0.30, and from 0.20 to 0.52 when using gut microbiota composition. Overall prediction accuracy (averaged over traits and time points) of phenotypes was 0.24 and 0.33 for Model_FB, 0.27 and 0.19 for Model_M, and 0.40 and 0.35 for Model_FB_M for the across-breed and across-room scenarios, respectively. This study shows how feeding behavior and gut microbiota composition provide non-redundant information in predicting growth in swine.

## 1. Introduction

In recent years, the swine industry has switched its focus towards implementing precision feeding and management practices to make pork production more sustainable and provide high-quality protein products to an expanding population [1]. Feeding behavior offers needed information for producers to adapt management and make decisions due to its relationship with feed efficiency and performance [2], rearing environment [3], and animal health and welfare [4]. As a result, feeding behavior measures in pigs have become a popular source of information for precision feeding in breeding programs. Real-time monitoring techniques, such as radio-frequency identification systems [5] and video recording [6,7], have proven effective in capturing the daily feeding activity of group-housed pigs. A better understanding of interindividual variability in feeding behavior, and its relationship with economic traits, would aid in maximizing the feeding efficiency and growth performance of pigs. 

Gut microbiota, which refers to the entire microorganism community in the intestinal tracts, has been associated with host homeostasis and physiological activities through close interaction with the central nervous system [8,9]. Numerous studies in humans and rodents have demonstrated the importance of the gut microbiome in predicting the development of diseases and its role as a critical biomarker in clinical diagnosis [10,11,12]. Fortunately, the rapid development of the next-generation sequencing technique improves the accessibility to 16S rRNA gene sequencing or shotgun sequencing data with a lower cost and more choices in sequencing depth for metagenomic research [13]. Recent work in swine has focused on characterizing the longitudinal variability of gut microbiota composition [14] and its association with host genome [15], feed efficiency [16], fat deposition [17], growth performance [18], and feeding behavior [19]. Furthermore, a growing number of studies have demonstrated that the gut microbiota is sensitive to changes in ambient temperature [20,21,22]. Thus, the gut microbiota can be a powerful tool to better understand and improve performance traits and a good marker for the interaction between animals and the environment. 

Nonetheless, the roles of feeding behavior and gut microbiota composition in maximizing growth performance in pigs have not been fully understood, particularly with data collected at several growth stages. Therefore, the objective of this study was to assess how feeding behavior and gut microbiome can predict finishing-stage growth and body composition traits across three popular pig breeds. Particularly, we aimed to examine the ability of feeding behavior and gut microbiome to predict phenotypes for different genetic backgrounds and rearing environments, allowing the better implementation of these two types of data in breeding and practical pork production. For this purpose, we employed two cross-validation strategies: leave-one-breed-out and leave-one-room-out, to assess the performance of predictive models. Prior to the prediction, we estimated the proportion of phenotypic variance attributed to the differences in feeding behavior and gut microbiota composition among animals, suggesting that they can be considered a significant predictor for the traits. Furthermore, we included feeding behavior and gut microbiota sequentially in the models free from other factors in the experimental design, aiming to provide more information on the prediction in situations where systematic data are limited or unavailable. 

## 2. Materials and Methods

### 2.1. Animals and Data

The phenotypic information used in this study is part of an existing dataset that was provided by Smithfield Premium Genetics (SPG; Rose Hill, NC, USA). Animal use approval was not needed for this study since all the data were collected from animals raised under the farm routine by Smithfield Premium Genetics (SPG; Rose Hill, NC, USA). Detailed descriptions of animals, family relationships, weaning age, diets, feeding behavior and microbiome data can be found in our previous publication [19]. Briefly, 651 purebred boars were employed, from either Duroc (DR; *n* = 205), Landrace (LR; *n* = 226), or Large White (LW; *n* = 220). After weaning, pigs were moved to nurseries and then finishers. In finishers, pigs were grouped by breed and housed in pens with an average of ten pigs per pen for the feeding trial. A single-space Feed Intake Recording Equipment (FIRE) feeder (Osborne Industries Inc., Osborne, KS, USA) was installed in each pen to feed pigs. Feed consumption, feeder occupation time, and animal identification were recorded when a pig visited. The present study used the rectal swabs collected from each pig at the middle (S1: 123 ± 4 days of age) and the end (S2: 158 ± 4 days of age) of the feeding trial. Correspondingly, feeding records were divided into two periods: from 99 to 140 days of age and from 141 to 163 ± 6 days of age. The feeding records were subjected to data quality control using the categories outlined in [23]. Seven feeding behavior measures, namely, average daily feed intake (ADFI), average daily feeder occupation time (AOTD), average daily feeding rate (ADFR), average daily number of visits to the feeder (ANVD), average feed intake per visit (AFIV), average feeder occupation time per visit (AOTV), and average feeding rate per visit (AFRV), were calculated for each pig during the given period, as described in our previous publications [2,19]. This study included the growth and body composition phenotypic records, including body weight (BW), backfat thickness (BF), loin depth (LD), and intramuscular fat (IMF) content, which were measured on live pigs when they reached market weight (120 kg), as described in [24]. An Aloka 500 Ultrasound device (Corometrics Medical Systems, Wallingford, CT, USA) was used to take photographs across the last three ribs to measure the BF, LD, and IMF. One measurement was obtained from each animal for each trait. Figure S1 depicts an overview of the experimental design.

### 2.2. 16S rRNA Gene Sequencing and Data Processing

The procedures for DNA extraction and purification for Illumina library preparation are described in [19,25]. Briefly, purified DNA pools were sequenced using the Illumina MiSeq platform to target the V4 region of the 16S rRNA gene in the DNA Sequencing Innovation Lab at the Center for Genome Sciences and Systems Biology of Washington University (St. Louis, MO, USA). The raw paired-end 250 bp sequences were processed according to the procedures described in our previous publications [19,25]. An amplicon sequence variant (ASV) feature table was generated. The ASVs were filtered out if their prevalence rate was less than 0.05 and their counts were fewer than 1000 across all samples for the given sampling point. After quality control, 724 and 824 ASVs remained for S1 and S2, respectively. 

### 2.3. Statistical Analysis

#### 2.3.1. Estimation of Variance Components and Microbiability

We fitted the following seven models to estimate the proportion of variance in growth and body composition phenotypes attributed to differences in host feeding behavior and gut microbiota composition among animals. Models were run on all animals using the data collected at each time point for each trait. We used Model_FB, Model_M, and Model_FB to estimate the variance components of feeding behavior and gut microbiota, demonstrating whether either of them can be considered a good predictor of traits. In addition, we fitted Model_S_P, Model_S_P_FB, Model_S_P_M, and Model_S_P_FB_M with the presence of family (sire) and group (pen) information to control the differences among genetic backgrounds and environments. The results of these four models were compared to previous ones to see if feeding behavior or gut microbiota composition can provide substitute information to the model when the systematic information is limited or unavailable. 

Model_FB:

(1) $$y_{kl}=\mu +fb_{k}+e_{kl}$$

Model_M:

(2) $$y_{kl}=\mu +m_{k}+e_{kl}$$

Model_FB_M:

(3) $$y_{kl}=\mu +fb_{k}+m_{k}+e_{kl}$$

Model_S_P:

(4) $$y_{ijk}=\mu +pen_{i}+sire_{j}+e_{ijk}$$

Model_S_P_FB:

(5) $$y_{ijkl}=\mu +pen_{i}+sire_{j}+fb_{k}+e_{ijkl}$$

Model_S_P_M:

(6) $$y_{ijkl}=\mu +pen_{i}+sire_{j}+m_{k}+e_{ijkl}$$

Model_S_P_FB_M:

(7) $$y_{ijkl}=\mu +pen_{i}+sire_{j}+fb_{k}+m_{k}+e_{ijkl}$$

In those models, y was the given trait, μ was the overall intercept, $pen_{i}$ was the random group effect of the *i*th pen, $sire_{j}$ was the random family effect of the *j*th sire, $fb_{k}$ was the random feeding behavior effect for individual *k* with fb ~ N (0, $F\sigma_{fb}^{2}$), where F is the linear kernel matrix of pairwise similarity between animals based on the seven feeding behavior measures and $\sigma_{fb}^{2}$ was the feeding behavior variance, $m_{k}$ was the random microbial effects for individual *k* with m ~ N (0, $M\sigma_{m}^{2}$), where M was the microbial relationship matrix and $\sigma_{m}^{2}$ was the microbial variance. Pen, sire, and residuals effects were assumed to be normally distributed as N(0, $I\sigma_{p}^{2}$), N(0, $I\sigma_{s}^{2}$), and N(0, $I\sigma_{e}^{2}$), respectively. 

We used the methods described by Tiezzi et al. and Ross et al. [26,27] to construct F and M matrices, respectively. Briefly, we started from a feeding behavior matrix with *n* by *b* dimensions, where *n* is the number of animals (*n* = 651) and *b* is the feeding behavior measures (*b* = 7), and an ASV abundance matrix with *n* by *p* dimensions, where *n* is the number of animals (*n* = 651) and *p* is the number of ASVs (*p* = 724 for S1; *p* = 824 for S2). Elements in the feeding behavior matrix or ASV abundance matrix were first log-transformed. A value of one was added to each element in the ASV matrix before the log-transformation for positive definiteness. Then, each variable in the feeding behavior matrix or ASV abundance matrix was centered and scaled to mean equal to zero and variance equal to a unit, resulting in two matrices $X_{m}$ or $X_{fb}$ for microbiota and feeding behavior, respectively. Lastly, the square F and M matrices with *n* by *n* dimensions were computed as F = $\frac{1}{b}X_{fb}X_{fb}^{T}$ and M = $\frac{1}{p}X_{m}X_{m}^{T}$, respectively.

The best linear unbiased prediction (BLUP) models with each matrix were fitted using the BGLR package [28] in the R software. The analyses were carried out using a Markov chain Monte Carlo (MCMC) algorithm with 150,000 iterations, 50,000 iterations discarded as burn-in, and 10 iterations set as the thinning interval. Model convergence was checked visually by inspecting the trace plots for each parameter posterior distribution and confirmed using the *geweke.diag* function with the default settings in the CODA package [29] in the R environment. 

The proportion of phenotypic variance attributed to the feeding behavior (fb^2^) or microbiota composition (*m*^2^, microbiability defined by Difford et al. [30]) was calculated for each model as the $\sigma_{fb}^{2}$ or $\sigma_{m}^{2}$ over the total phenotypic variance.

$$fb^{2}=\frac{\sigma_{fb}^{2}}{\sigma_{fb}^{2}+\left(\sigma_{m}^{2}\right)+\sigma_{e}^{2}\;}$$

$$m^{2}=\frac{\sigma_{m}^{2}}{\sigma_{m}^{2}+\left(\sigma_{fb}^{2}\right)+\sigma_{e}^{2}\;}$$

#### 2.3.2. Predictive Ability of Microbiota Composition and Feeding Behavior

To better assess the predictive ability of feeding behavior and gut microbiota, we used Model_FB, Model_M, and Model_FB_M without interferences of systematic effects as previously described to make the prediction. The leave-one-breed-out and leave-one-room-out cross-validation strategies were applied to assess the prediction performance across rearing environments and genetic backgrounds from feeding behavior and gut microbiota. For the across-breed scenario, two of the three breeds were used as the training set, and the remaining one as the validation set. The predictions were repeated three times until every breed was considered. Similarly, seven of the eight rooms were used as the training set to predict the remaining one in the across-room scenario. The models were implemented using the BGLR package [28] in the R environment with the same setting as described in the previous section. Prediction accuracy was calculated as the Pearson’s correlations between predicted and observed phenotypes in the validation set. Furthermore, the mean squared errors (MSE) were calculated as the average of the squares of the differences between predicted and observed phenotypes in the validation set to evaluate the predictive performance. 

#### 2.3.3. Post-Analysis of fb^2^ and m^2^ Estimates and Prediction Accuracy

We performed a post-analysis to compare the fb^2^ and m^2^ estimates across levels of traits, models, or time points by fitting ANOVA with the PROC GLM in SAS (v9.4, SAS Institute, Carry, NC, USA). Similarly, we used ANOVA to compare prediction accuracy across levels of the factors in the experimental design. The regression models listed below were fitted. 

For the prediction using leave-one-breed-out cross-validation:

(8) $$y_{ijklm}=B_{i}+T_{j}+Tr_{k}+M_{l}+BT_{ij}+BTr_{ik}+BM_{il}+TTr_{jk}+TM_{jl}+TrM_{kl}+e_{ijklm}$$

For the prediction using leave-one-room-out cross-validation:

(9) $$y_{ijklm}=R_{i}+T_{j}+Tr_{k}+M_{l}+RT_{ij}+RTr_{ik}+RM_{il}+TTr_{jk}+TM_{jl}+TrM_{kl}+e_{ijklm}$$

where $y_{ijklm}$ is the predictive accuracy of each breed(room)/time/trait/model combination; $B_{i}$ is the fixed effects of breed (3 levels: DR, LR, and LW); $R_{i}$ is the fixed effects of room (8 levels: room 1 to 8); $T_{j}$ is the fixed effects of time point for microbiome and feeding behavior measurements (2 levels: S1 and S2); $M_{l}$ is the fixed effects of model (3 levels: Model_FB, Model_M, and Model_FB_M); $B\left(R\right)T_{ij}$, $B\left(R\right)Tr_{ik}$, $B\left(R\right)M_{il}$, $TTr_{jk}$, $TM_{jl}$, and $TrM_{kl}$ are the pairwise interactions of the main effects; and $e_{ijklm}$ is the residual assumed N(0, $I\sigma_{e}^{2}$). The least-squares means, and their contrasts, were obtained with the LSMEANS statement with the Tukey option in the PROC GLM. A *p*-value less than or equal to 0.05 was considered significant.

## 3. Results

### 3.1. Data Summary

Table S1 shows descriptive statistics for growth and body composition traits measured on finishing pigs. Statistics of feeding behavior measures during the two periods can be found in [19]. Gut microbial compositions of the three breeds were previously reported by Bergamaschi et al. [24].

### 3.2. Proportion of Phenotypic Variances Explained by Feeding Behavior and Microbiota Composition

**Figure 1:**
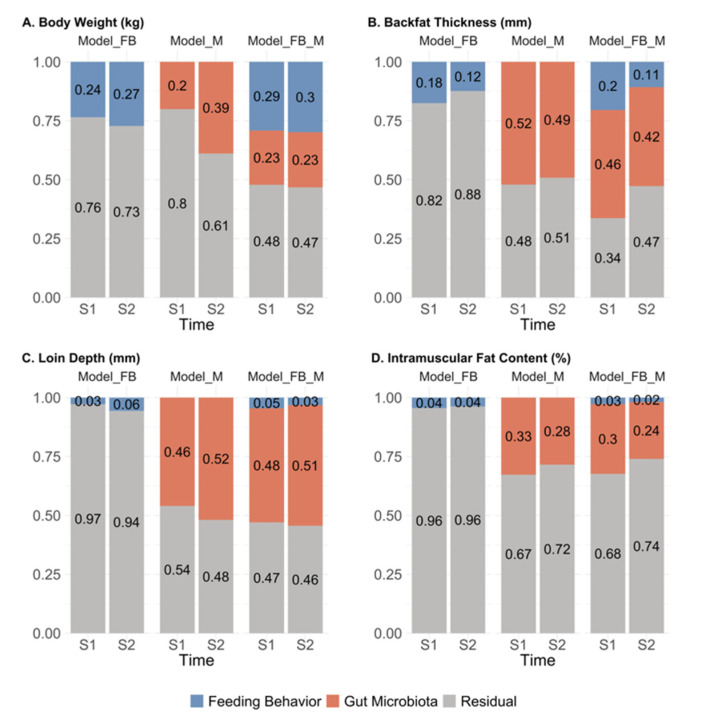
Proportion of total phenotypic variance explained by feeding behavior, gut microbiota composition, or residual in Model_FB, Model_M, and Model_FB_M by trait and sampling time point. Colors represent the proportion of phenotypic variance explained by feeding behavior (blue), microbiota (red), or residual (grey). The x-axis represents two sampling time points (S1 and S2). The proportion value is indicated on the y-axis. (**A**) Proportion estimates for body weight (kg); (**B**) Proportion estimates for backfat thickness (mm); (**C**) Proportion estimates for loin depth (mm); (**D**) Proportion estimates for intramuscular fat content (%). S1: sampling time point 1; S2: sampling time point 2.

The proportions of phenotypic variance attributed to feeding behavior and gut microbiota composition estimated by Model_FB, Model_M, and Model_FB_M are shown in Figure 1. Across traits and data collection time points, the proportion of phenotypic variance explained by feeding behavior ranged from 0.03 to 0.27 for Model_FB and from 0.02 to 0.30 for Model_FB_M, while the microbiability estimates ranged from 0.20 to 0.52 for Model_M and from 0.23 to 0.51 for Model_FB_M. The proportion of phenotypic variance attributed to feeding behavior or gut microbiota composition varied depending on the trait considered. Feeding behavior explained a small to moderate proportion of the variance in BW and BF, ranging from 0.11 to 0.30, but little for LD and IMF. In the two models with microbiota, the microbiability estimates of BF and LD were greater than those of BW and IMF. Minor differences in the proportion estimates were observed between the two data collection time points. In most cases, we found little differences in the proportions of phenotypic variance explained by feeding behavior or microbiota composition between Model FB_M and Model_FB or Model_M. 

We added the pen and sire effects to the models and investigated how the variance components changed when including systematic effects (Figure S2). We observed an increase in the proportion of variance explained by the pen when comparing the estimates obtained by the model with only pen and sire effects (Model_S_P) to the model also including feeding behavior (Model S_P_M), implying that there may be collinearity between the feeding behavior and pen factors for BW and BF traits. Furthermore, when we added feeding behavior to the model for the same two traits, we noticed a decrease in the proportion estimates for sire and microbiota effects. In contrast, all the variables provided non-redundant information to the models for LD and IMF. Table S2 shows the mean and standard error of the proportion estimates in different models. 

**Figure 2:**
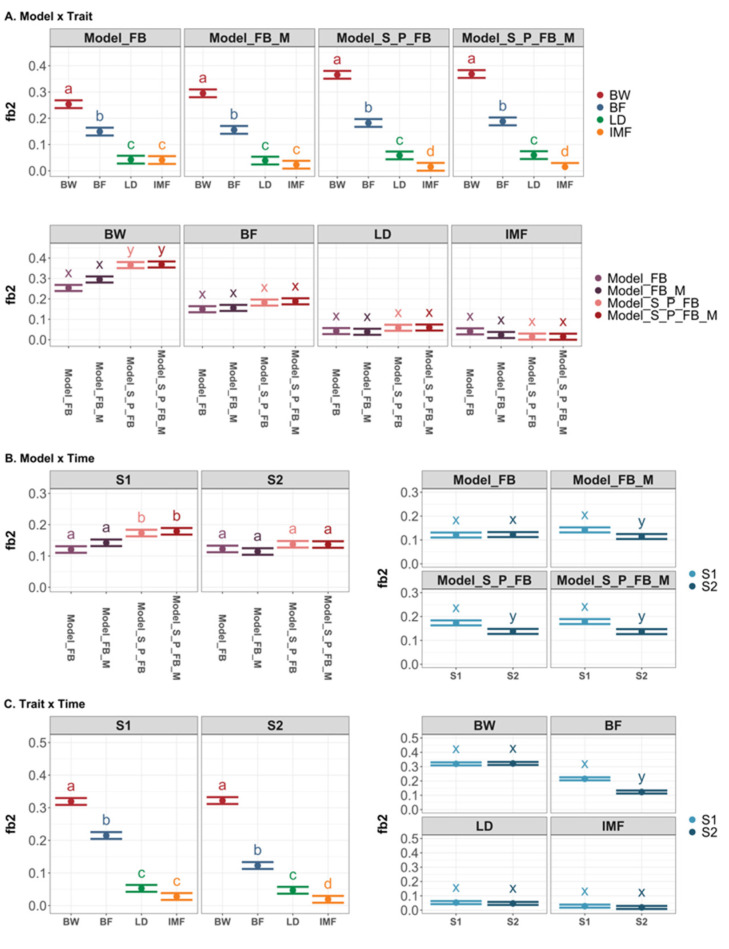
Least square means of the proportion in phenotypic variance explained by feeding behavior (fb^2^) with 95% confidence interval and contrasts among levels of significant two-way interaction effects. Different letters denote *p* < 0.05 for each level of the factor interested. (**A**) LSmeans with 95% CI and contrast among levels of interaction effects between model and trait; (**B**) LSmeans with 95% CI and contrast among levels of interaction effects between model and time; (**C**) LSmeans with 95% CI and contrast among the levels of interaction effects between trait and time. BW: body weight; BF: backfat thickness; LD: loin depth; IMF: intramuscular fat. S1: sampling time point 1; S2: sampling time point 2.

Figure 2 depicts the post-analysis results on the proportion of phenotypic variance explained by feeding behavior (fb^2^) across four traits, two data collection time points, and four models with feeding behavior effects. The effect of the two-way interaction between model, trait, and time on fb^2^ estimates was significant. Regardless of the model fitted, the fb^2^ estimates were highest for BW, followed by BF, LD, and IMF. However, the model with pen and sire effects had higher fb^2^ estimates than other models for BW, while the estimates for different traits were similar across models (Figure 2A). Using the data collected at S1, the models with pen and sire effects (Model_S_P_FB and Model_S_P_FB_M) had higher fb^2^ estimates than the models without those effects (Model_FB and Model_FB_M). Still, no difference was found between models using the data collected at S2 (Figure 2B). At two data collection time points, the rankings of fb^2^ estimates among four traits were similar. The feeding behavior collected around S1 was more informative in explaining the variance in BF than the data collected around S2 (Figure 2C). 

**Figure 3:**
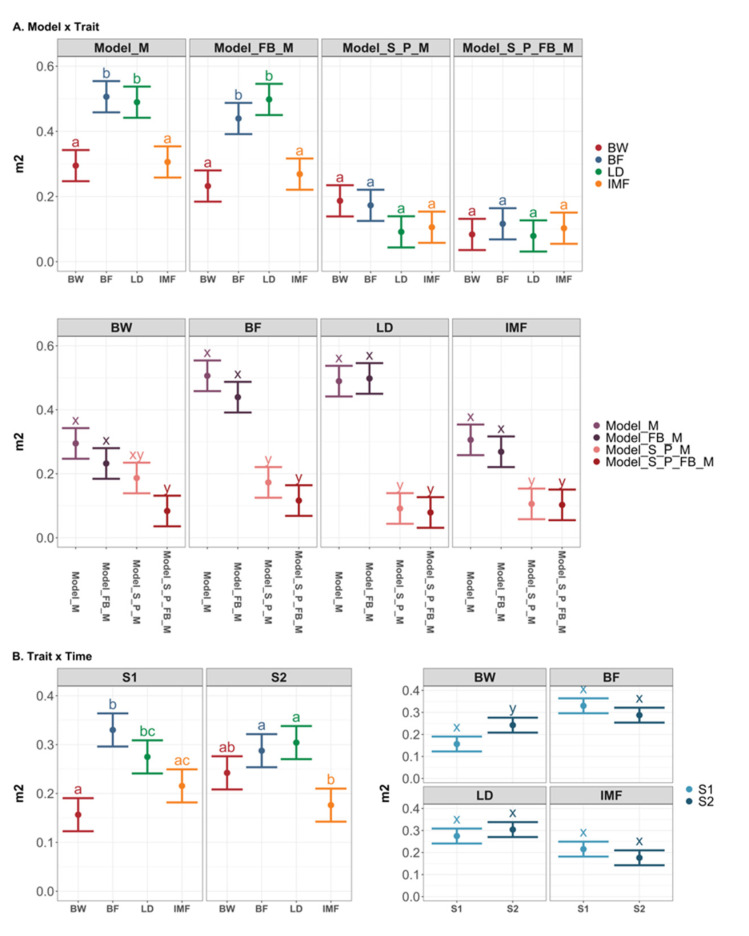
Least square means of microbiability (m^2^) with 95% confidence interval and contrasts among levels of significant two-way interaction effects. Different letters denote *p* < 0.05 for each level of the factor interested. (**A**) LSmeans with 95% CI and contrast among levels of interaction effects between model and trait; (**B**) LSmeans with 95% CI and contrast among the levels of interaction effects between trait and time. BW: body weight; BF: backfat thickness; LD: loin depth; IMF: intramuscular fat. S1: sampling time point 1; S2: sampling time point 2.

Figure 3 depicts the results of the post-analysis on m^2^. The two-way interactions between model and trait, and time and trait, were significant. The m^2^ estimates for BF and LD in Model_M and Model_FB_M were higher than BW and IMF traits but similar across traits in the models with pen and sire effects. Furthermore, the m^2^ estimates were higher in the Model_M and Model_FB_M than in the models including pen and sire effects for all traits (Figure 3A). Except for BW, the gut microbiome sampled at two time points provided similar information to the models in estimating m^2^ (Figure 3B). 

### 3.3. Across-Breed Prediction Performance

To assess the predictive ability of host feeding behavior and gut microbiota composition in situations where systematic information is limited or unavailable, we fitted Model_FB, Model_M, and Model_FB_M to predict host phenotypes using leave-one-breed-out and leave-one-room-out cross-validation strategies. 

**Figure 4:**
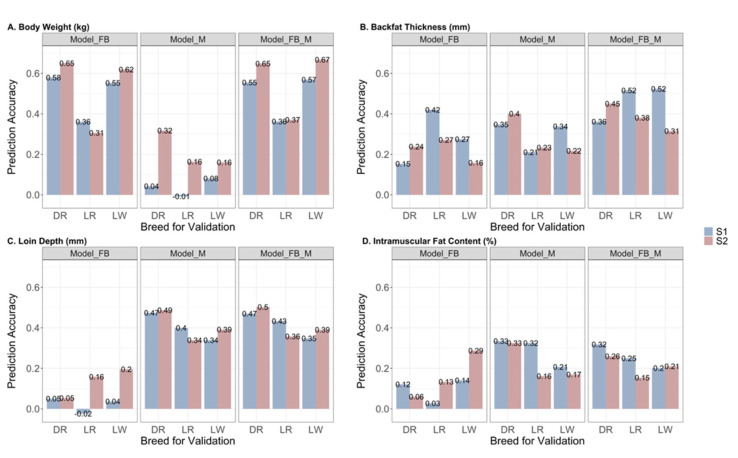
Prediction accuracy of Model_FB, Model_M, and Model_FB_M for each across-breed prediction scenario by trait and sampling time point. Colors represent two time points for data collection: S1 (blue) and S2 (red). The x-axis indicates the breed used as the validation in the prediction. The y-axis is the prediction accuracy, which is the correlation value between predicted and observed phenotypes. (**A**) Prediction accuracy for body weight (kg); (**B**) Prediction accuracy for backfat thickness (mm); (**C**) Prediction accuracy for loin depth (mm); (**D**) Prediction accuracy for intramuscular fat content (%). DR: Duroc; LR: Landrace; LW: Large White. S1: sampling time point 1; S2: sampling time point 2.

The prediction accuracy obtained by the three models using the leave-one-breed-out cross-validation strategy is shown in Figure 4. The feeding behavior best predicted BW, with an accuracy ranging from 0.31 to 0.65, followed by BF, with an accuracy ranging from 0.15 to 0.42. The prediction accuracy obtained by Model_M ranged from −0.01 to 0.32 for BW, 0.21 to 0.35 for BF, 0.34 to 0.49 for LD, and 0.16 to 0.33 for the IMF trait across two sampling points. The inclusion of both feeding behavior and gut microbiota in Model_FB_M improved overall accuracy in predicting BW and BF but had no addictive effects in predicting LD and IMF, compared to Model_FB or Model_M. Regardless of the model and the prediction scenario, the accuracy ranged from −0.01 to 0.58 and 0.16 to 0.67 for BW, from 0.15 to 0.52 and 0.16 to 0.45 for BF, from −0.02 to 0.47 and 0.05 to 0.50 for LD, and from 0.03 to 0.33 and 0.06 to 0.33, for IMF using the data collected at S1 and S2 in the prediction, respectively. 

The MSE obtained by the three models using the leave-one-breed-out cross-validation strategy is summarized in Table 1. With smaller MSE values averaged over the three scenarios across breeds, Model_FB better predicted BW and BF, whereas Model_M had better performance predicting LD and IMF. When both predictors were included in the model, the MSE was lower in predicting BW and LD using data collected from both time points and BF and IMF using data collected from S1, compared to models with only one predictor. Consistently across the three models, data collected at S2 better informed the models for predicting BW than S1. Compared to other scenarios for the same trait, the feeding behavior and gut microbiota of DR and LW pigs measured at two time points better predicted the BF of LR pigs. A similar result was found when predicting the LD of DR pigs using the information of LR and LW pigs. However, the prediction for BW of LR pigs from the data of the other two breeds had lower accuracy and lower MSE compared to different scenarios. This inconsistency between accuracy and MSE estimates was nuanced, but it must be interpreted with extra caution.

**Figure 5:**
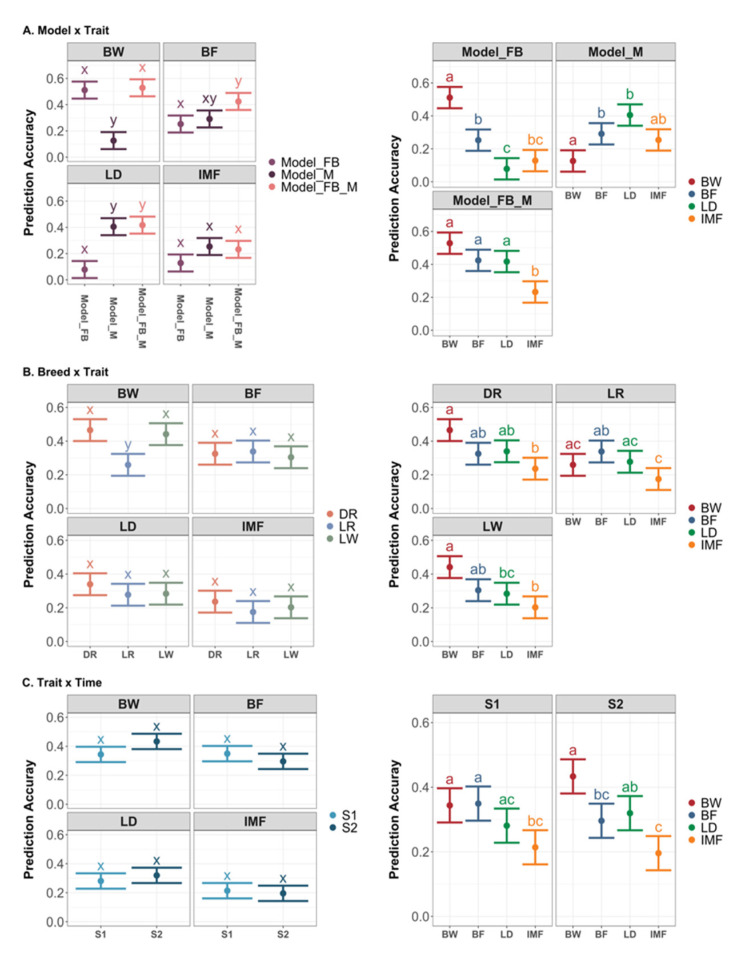
Least square means of prediction accuracy with 95% confidence interval and contrasts among levels of significant two-way interaction effects in the across-breed prediction. Different letters denote *p* < 0.05 for each level of the factor interested. (**A**) LSmeans with 95% CI and contrast among levels of interaction effects between model and trait; (**B**) LSmeans with 95% CI and contrast among levels of interaction effects between breed in the validation and trait; (**C**) LSmeans with 95% CI and contrast among levels of interaction effects between trait and time. DR: Duroc; LR: Landrace; LW: Large White. BW: body weight; BF: backfat thickness; LD: loin depth; IMF: intramuscular fat. S1: sampling time point 1; S2: sampling time point 2.

We performed a post-analysis on prediction accuracy and compared the estimates among levels of the model, trait, time point, scenario, and the two-way interactions between these factors. The LSmeans with 95% confidence intervals and significant contrasts are depicted in Figure 5. Three models predicted BF and IMF with comparable accuracy (Figure 5A). The models with feeding behavior outperformed Model_M in the BW prediction, with a mean accuracy of 0.51 for Model_FB, 0.53 for Model_FB_M, and 0.13 for Model_M. By comparison, the gut microbiota predicted LD more accurately than Model_FB, with a mean accuracy of 0.41 for Model_M, 0.42 for Model_FB_M, and 0.08 for Model_FB. Except for predicting the BW using the information of the other two breeds, there was no significant difference in prediction accuracy across the three prediction scenarios for the given trait (Figure 5B). In addition, there was no significant difference in the accuracy between the predictions using the data collected at S1 and S2 (Figure 5C). 

### 3.4. Across-Room Prediction Performance

We also investigated how feeding behavior and gut microbiota can be used to predict phenotypes among environments. Table 2 shows the prediction accuracy and MSE in terms of means and standard deviations over the eight folds of the leave-one-room-out cross-validation scenario. Overall, the predictive abilities of Model_FB, Model_M, and Model_FB_M were comparable to the previous results of the across-breed prediction. Across models and sampling time points, the prediction accuracy ranged from 0.16 to 0.60 for BW, 0.33 to 0.61 for BF, 0.11 to 0.26 for LD, and −0.02 to 0.12 for IMF. We found variations in accuracy and MSE estimates when predicting the phenotypes of pigs raised in different rooms. The largest standard deviation of accuracy was 0.17 for Model_FB, 0.13 for Model_M, and 0.16 for Model_FB_M in predicting BW using the data collected at S1. In comparison, the smallest standard deviation was 0.09 for Model_FB predicting IMF, and 0.07 for Model_M and Model_FB_M predicting LD across the eight scenarios. When the three models were compared, Model_FB_M had the lowest mean MSE in predicting BW and BF, whereas Model_M had the lowest mean MSE in predicting LD and IMF and the smallest standard deviation across eight scenarios in predicting BW, BF, and LD of pigs. 

**Figure 6:**
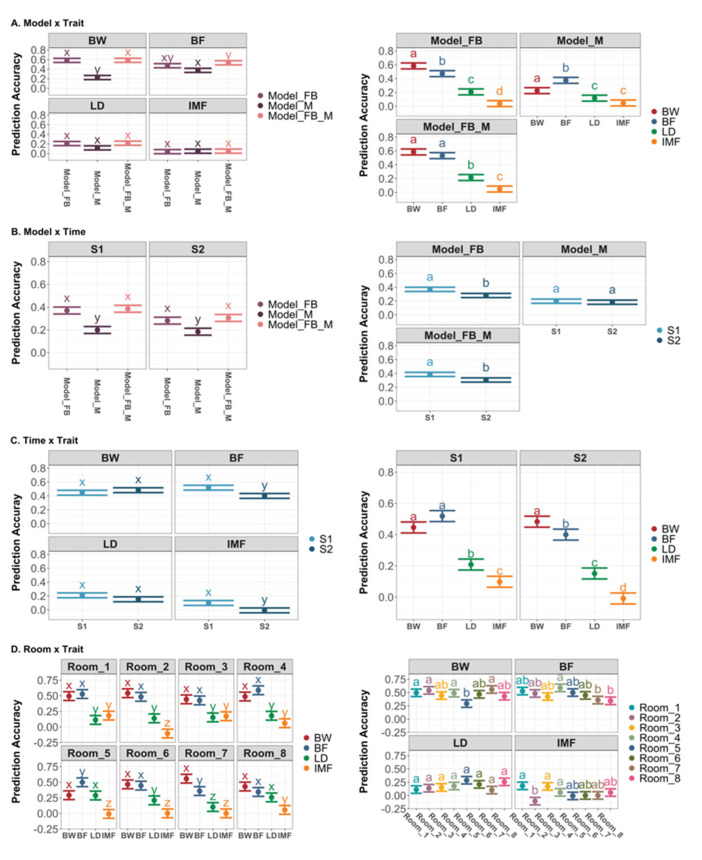
Least square means of prediction accuracy with 95% confidence interval and contrasts among levels of significant two-way interaction effects in the across-room prediction. Different letters denote *p* < 0.05 for each level of the factor interested. (**A**) LSmeans with 95% CI and contrast among levels of interaction effects between model and trait; (**B**) LSmeans with 95% CI and contrast among levels of interaction effects between model and time; (**C**) LSmeans with 95% CI and contrast among levels of interaction effects between trait and time; (**D**) LSmeans with 95% CI and contrast among levels of interaction effects between room in the validation and trait. DR: Duroc; LR: Landrace; LW: Large White. BW: body weight; BF: backfat thickness; LD: loin depth; IMF: intramuscular fat. S1: sampling time point 1; S2: sampling time point 2.

Figure 6 depicts the results of significant two-way interaction effects on prediction accuracy in the post-analysis. The interaction effects between model and trait, and time and trait, on prediction accuracy were similar to those for leave-one-breed-out prediction. Regardless of which room was targeted in the prediction, the patterns of prediction accuracy for the four traits were similar. There were minor differences in prediction accuracy for BW, BF, and IMF, but no difference for LD across the eight scenarios targeting different rooms. 

## 4. Discussion

In this study, rather than studying the individual effects of feed intake, feeder occupation time, feeding rate, and the number of visits to the feeder on the traits, we fitted feeding behavior as a whole predictor in the model to estimate its contribution in explaining the proportion of phenotypic variance. We found a small to moderate proportion of variance in body weight and backfat thickness of finishing pigs associated with variation in feeding behavior in the scenarios with and without pen and sire effects controlled in the model. Similar to the findings of this study, Rauw and colleagues found that feeding frequency, feeding duration, feeding rate, and feed intake were significantly related to growth and fat deposition after pen effects were pre-adjusted [31]. Another study also found that feeding rate, feed intake, and time in eating were all highly correlated with the growth of pigs [32]. Results suggest collinearity between the effects of pen and feeding behavior on body weight and backfat thickness. It can be explained by differences in feeding behavior between pens due to host genetics, social ranks among animals in pen, or other environmental factors [33,34]. In future studies, a larger sample size will be required to control this collinearity in the model and investigate the effects of other factors on the feeding behavior of group-housed pigs.

Furthermore, feeding behavior did not contribute to the variance in loin depth in our study, which is consistent with the findings in a previous study [32]. We also found overlapping in the phenotypic variance of body weight and backfat thickness explained by feeding behavior and sire. The results were expected given that feeding behavior measures are heritable, with moderate heritability estimated by Do et al. in three pig breeds [35]. Feeding behavior and gut microbiota composition exhibited similar overlapping effects in explaining body weight and backfat thickness variance. It can be explained by the small to moderate associations between feeding behavior and gut microbiota composition addressed in our previous study [19]. 

The microbiability quantified the overall relationship between gut microbiota composition and phenotypes, accounting for alpha and beta diversity [36]. Our estimates of microbiability for backfat thickness, loin depth, and intramuscular fat content obtained from the model with pen and sire effects are comparable to the microbiability reported by Piush et al. for the same traits measured on the carcass of crossbred pigs [37]. To the best of our knowledge, we are the first to estimate microbiability for the body weight of finishing pigs. The gut microbiota composition accounted for 12% to 25% of the variance in body weight at two sampling time points in the model that controlled for pen and sire effects. A previous study reported a microbiability of 28% for daily gain in German Piétrain sows [38]. As expected, when pen and sire information was not present, the gut microbiota composition better informed the model and accounted for a greater proportion of phenotypic variance. Numerous studies have highlighted the effects of environmental factors on shaping the gut microbiota composition in both humans and animals [39,40,41].

Moreover, the gut microbiota can be regarded as a sensitive indicator that rapidly responds to environmental changes or stressors in various animal species [42,43,44,45]. Thus, it is likely that differences in the gut microbiota composition of animals kept in different pens partially represented the pen effects on the traits, particularly on loin depth, in our study. These findings suggest that when systematic information about the rearing environment is unavailable or limited, the gut microbiota may be a valuable source of information for understanding and improving complex traits in swine. However, more research is required to address this issue. 

We fitted three models using leave-one-breed-out and leave-one-room-out cross-validation schemes to assess the ability of feeding behavior, gut microbiota composition, or both to predict traits for different breeds and environments. The outcomes differed depending on the trait considered in the prediction. Feeding behavior outperformed gut microbiota composition in predicting body weight in both scenarios. Furthermore, including both predictors in the model improved accuracy when predicting backfat thickness and decreased MSE when predicting body weight. These findings suggest that feeding behavior and gut microbiota can provide non-redundant information to the prediction model for those traits. Predicting the body weight of Landrace pigs from the feeding behavior of Duroc and Large White pigs was more challenging than other scenarios. Aside from that, the accuracy for the body composition traits was comparable across scenarios in which different breeds were considered in the prediction. We observed a slight variation in prediction accuracy across scenarios for the given trait in the across-room forecast. According to our findings, feeding behavior and gut microbiota performed well for predictions made across genetic backgrounds and environments. Overall, predictions from feeding behavior had a higher accuracy for the across-room than across-breed scenario, whereas the opposite pattern was found for predictions from gut microbiota composition. In this case, the environment may have a more significant influence on gut microbiota composition than host genetics, making across-environment predictions from the microbiome more challenging. We believe that this should be further studied on a larger number of animals. In addition, we found some negative prediction accuracy values close to zero, which may be attributed to the use of leave-one-out cross-validation to evaluate predictive performance. In such cases, the prediction accuracy can be taken as zero. 

In addition, we compared the predictive performance of data collected at two growth stages. Overall, the results regarding data collection time were consistent between the across-breed and across-room scenarios, but they were highly dependent on the trait. Regardless of feeding behavior or gut microbiota, data collected later were more useful in predicting body weight but less informative in predicting backfat thickness and intramuscular fat content than the data collected earlier in the study. Maltecca et al. suggested that the microbiome sampled at the middle stage of growth provided more information for the model to predict growth and carcass traits of crossbred pigs [46]. However, the swine gut microbiome is dynamic over time [47,48,49], making microbial prediction more difficult. Modeling longitudinal microbiome data along growth would be of interest in future research to better investigate how this dynamic pattern of the gut microbiome is associated with pig growth performance. In addition, the results would be more robust if the prediction was made on specific microbes that are significantly correlated to the trait. 

## 5. Conclusions

In summary, this study investigated the ability of feeding behavior and gut microbiome in predicting growth and body composition traits of finishing pigs across breeds and rearing environments. Our findings suggest that the predictive performance of feeding behavior and gut microbiota differed depending on the trait and scenario studied. The feeding behavior outperformed gut microbiota composition in predicting body weight in both scenarios, whereas the gut microbiota composition better predicted the loin depth compared to feeding behavior in the across-breed prediction. The inclusion of feeding behavior and gut microbiota composition in the model improved backfat thickness prediction. The findings in the current study highlight the critical roles of feeding behavior and gut microbiome in understanding and improving pig growth performance. 

## Figures and Tables

**Table 1:** Mean squared error of prediction using leave-one-breed-out cross-validation.

Trait	Time	Training ^1^	Validation	Model		
				Model_FB	Model_M	Model_FB_M
Body Weight (kg)	S1	LR + LW	DR	126.00	142.35	107.76
		DR + LW	LR	115.17	118.03	118.87
		DR + LR	LW	117.99	156.86	106.92
		Average		119.72	139.08	111.18
	S2	LR + LW	DR	107.83	127.53	95.03
		DR + LW	LR	110.10	117.06	105.61
		DR + LR	LW	112.49	148.73	98.36
		Average		110.14	131.10	99.67
Backfat Thickness (mm)	S1	LR + LW DR + LW	DR LR	13.75 7.24	19.30 11.98	15.78 8.09
		DR + LR	LW	16.08	15.42	11.96
		Average		12.36	15.57	11.94
	S2	LR + LW	DR	11.09	18.12	14.59
		DR + LW	LR	8.26	10.97	9.47
		DR + LR	LW	17.63	17.48	16.04
		Average		12.33	15.52	13.37
Loin Depth (mm)	S1	LR + LW	DR	45.14	34.22	34.01
		DR + LW	LR	47.66	39.09	38.74
		DR + LR	LW	51.18	42.95	42.37
		Average		47.99	38.75	38.37
	S2	LR + LW	DR	45.17	36.56	33.00
		DR + LW	LR	45.40	43.68	43.84
		DR + LR	LW	48.54	44.66	42.44
		Average		46.37	41.63	39.76
Intramuscular Fat Content (%)	S1	LR + LW	DR	0.77	0.76	0.74
		DR + LW	LR	0.60	0.51	0.53
		DR + LR	LW	0.59	0.60	0.59
		Average		0.65	0.62	0.62
	S2	LR + LW	DR	0.80	0.72	0.73
		DR + LW	LR	0.58	0.58	0.59
		DR + LR	LW	0.57	0.63	0.62
		Average		0.65	0.64	0.65

^1^ DR: Duroc; LR: Landrace; LW: Large White.

**Table 2:** Accuracy and MSE of the across-room prediction using the three models by trait and time.

Trait ^1^	Time	Model_FB		Model_M		Model_FB_M	
		r	MSE	r	MSE	r	MSE
BW (kg)	S1	0.60 ± 0.17	111.25 ± 38.74	0.16 ± 0.13	132.15 ± 18.06	0.58 ± 0.16	109.56 ± 38.24
	S2	0.57 ± 0.13	103.01 ± 22.24	0.29 ± 0.09	125.44 ± 14.77	0.59 ± 0.11	101.34 ± 24.49
BF (mm)	S1	0.54 ± 0.13	10.11 ± 4.75	0.41 ± 0.10	9.40 ± 3.15	0.61 ± 0.11	7.77 ± 3.46
	S2	0.41 ± 0.11	10.51 ± 4.45	0.33 ± 0.10	9.84 ± 2.69	0.46 ± 0.09	9.32 ± 3.34
LD (mm)	S1	0.26 ± 0.12	55.35 ± 38.64	0.13 ± 0.13	51.63 ± 29.88	0.23 ± 0.11	52.37 ± 30.91
	S2	0.15 ± 0.11	53.65 ± 36.47	0.11 ± 0.07	47.57 ± 27.58	0.20 ± 0.07	49.23 ± 28.71
IMF (%)	S1	0.08 ± 0.09	0.71 ± 0.34	0.10 ± 0.12	0.67 ± 0.30	0.12 ± 0.13	0.68 ± 0.29
	S2	0.00 ± 0.14	0.70 ± 0.34	0.00 ± 0.09	0.73 ± 0.30	−0.02 ± 0.11	0.73 ± 0.30

^1^ Data are presented as mean ± SD over eight scenarios for each trait/model/time combination. BW: body weight (kg); BF: backfat thickness (mm); LD: loin depth (mm); IMF: intramuscular fat content (%). S1: sampling time point 1; S2: sampling time point 2.

## Data Availability

Publicly available 16S rRNA gene sequencing datasets were analyzed in this study. This data can be found in the NCBI repository, with the accession number PRJNA747026. Restrictions apply to the availability of phenotypic data. Data were obtained from Smithfield Premium Genetics (Rose Hill, NC, USA) and are available with the permission of Smithfield Premium Genetics (Rose Hill, NC, USA).

## Supplementary Materials

The following supporting information can be downloaded at: https://www.mdpi.com/article/10.3390/genes13050767/s1, Figure S1: Overall view of experimental design; Figure S2: Proportion of total phenotypic variance explained by pen, sire, feeding behavior, gut microbiota composition, or residual in Model_S_P, Model_S_P_FB, Model_S_P_M, and Model_S_P_FB_M by trait and sampling time point. Colors represent the proportion of phenotypic variance explained by pen (green), sire (yellow), feeding behavior (blue), microbiota (red), or residual (grey). The x-axis represents two sampling time points. The proportion value is indicated on the y-axis; (A) Proportion estimates for body weight (kg); (B) Proportion estimates for backfat thickness (mm); (C) Proportion estimates for loin depth (mm); (D) Proportion estimates for intramuscular fat content (%). Table S1: Descriptive statistics for growth and body composition traits measured on finishing pigs by breed. Table S2: Proportion of phenotypic variance explained by the pen, sire, feeding behavior, and gut microbiota composition in different models. Data are presented as the mean (SE).
