# Supplementary material for: Use of Host Feeding Behavior and Gut Microbiome Data in Estimating Variance Components and Predicting Growth and Body Composition Traits in Swine
Source: Genes (Basel). 2022 Apr 26;13(5):767. doi: 10.3390/genes13050767 (PMC9140470; doi:10.3390/genes13050767)
Supplement: Supplementary file 1 [file genes-13-00767-s001.zip › genes-1645048 -supplementary FINAL/genes-1645048-supplementary table S2.pdf]

Table S2. Proportion of phenotypic variance explained by the pen, sire, feeding behavior, and gut microbiota composition in different models. Data are presented as mean (SE)[a].

Trait	Time	Effect	Model FB	Model M	Model FB_M	Model S_P	Model S_P_FB	Model S_P_M	Model S_P_FB_M
BW	S1	P	-	-	-	0.15 (0.03)	0.25 (0.02)	0.14 (0.03)	0.22 (0.03)
		S	-	-	-	0.11 (0.03)	0.05 (0.01)	0.10 (0.03)	0.04 (0.01)
		FB	0.24 (0.03)	-	0.29 (0.03)	-	0.37 (0.02)	-	0.38 (0.02)
		M	-	0.20 (0.05)	0.23 (0.03)	-	-	0.12 (0.03)	0.07 (0.02)
	S2	P	-	-	-	0.15 (0.03)	0.18 (0.02)	0.12 (0.03)	0.16 (0.02)
		S	-	-	-	0.11 (0.03)	0.06 (0.01)	0.10 (0.03)	0.05 (0.01)
		FB	0.27 (0.03)	-	0.30 (0.03)	-	0.36 (0.03)	-	0.36 (0.03)
		M	-	0.39 (0.05)	0.23 (0.04)	-	-	0.25 (0.05)	0.10 (0.02)
BF	S1	P	-	-	-	0.22 (0.05)	0.32 (0.03)	0.16 (0.04)	0.21 (0.04)
		S	-	-	-	0.21 (0.05)	0.09 (0.02)	0.20 (0.04)	0.10 (0.02)
		FB	0.18 (0.03)	-	0.20 (0.02)	-	0.23 (0.02)	-	0.25 (0.03)
		M	-	0.52 (0.04)	0.46 (0.03)	-	-	0.20 (0.04)	0.14 (0.03)
	S2	P	-	-	-	0.22 (0.05)	0.31 (0.04)	0.21 (0.05)	0.31 (0.04)
		S	-	-	-	0.21 (0.05)	0.11 (0.03)	0.19 (0.04)	0.10 (0.03)
		FB	0.12 (0.02)	-	0.11 (0.02)	-	0.13 (0.02)	-	0.13 (0.02)
		M	-	0.49 (0.04)	0.42 (0.04)	-	-	0.15 (0.04)	0.09 (0.02)
LD	S1	P	-	-	-	0.49 (0.03)	0.51 (0.03)	0.44 (0.04)	0.47 (0.03)
		S	-	-	-	0.07 (0.02)	0.05 (0.01)	0.07 (0.02)	0.05 (0.01)
		FB	0.03 (0.01)	-	0.05 (0.01)	-	0.07 (0.02)	-	0.07 (0.02)
		M	-	0.46 (0.04)	0.48 (0.04)	-	-	0.08 (0.02)	0.07 (0.02)
	S2	P	-	-	-	0.49 (0.03)	0.51 (0.03)	0.45 (0.04)	0.47 (0.04)
		S	-	-	-	0.07 (0.02)	0.05 (0.01)	0.07 (0.02)	0.05 (0.01)
		FB	0.06 (0.02)	-	0.03 (0.01)	-	0.05 (0.01)	-	0.05 (0.01)
		M	-	0.52 (0.04)	0.51 (0.04)	-	-	0.10 (0.03)	0.09 (0.02)
IMF	S1	P	-	-	-	0.23 (0.04)	0.22 (0.04)	0.17 (0.04)	0.16 (0.04)
		S	-	-	-	0.07 (0.02)	0.06 (0.02)	0.07 (0.02)	0.07 (0.02)
		FB	0.04 (0.01)	-	0.03 (0.01)	-	0.02 (0.01)	-	0.02 (0.01)
		M	-	0.33 (0.04)	0.30 (0.04)	-	-	0.12 (0.03)	0.12 (0.03)

S2	P	-	-	-	0.23 (0.04)	0.21 (0.04)	0.20 (0.04)	0.18 (0.04)
	S	-	-	-	0.07 (0.02)	0.07 (0.02)	0.06 (0.02)	0.07 (0.02)
	FB	0.04 (0.01)	-	0.02 (0.01)	-	0.01 (0.01)	-	0.01 (0.01)
	M	-	0.28 (0.05)	0.24 (0.05)	-	-	0.09 (0.03)	0.09 (0.02)

[a] BW = body weight; BF = backfat thickness; LD = loin depth; IMF = intramuscular fat content; S1 = sampling time point 1; S2 = sampling time point 2; P = pen; S = sire; FB = feeding behavior; M = gut microbiota.
