# Supplementary material for: Use of Host Feeding Behavior and Gut Microbiome Data in Estimating Variance Components and Predicting Growth and Body Composition Traits in Swine
Source: Genes (Basel). 2022 Apr 26;13(5):767. doi: 10.3390/genes13050767 (PMC9140470; doi:10.3390/genes13050767)
Supplement: Supplementary file 1 [file genes-13-00767-s001.zip › genes-1645048 -supplementary FINAL/TableS1.pdf]

Table S1. Descriptive statistics for growth and body composition traits measured on finishing pigs by breed.

Breed	Statistics	Off-test Trait			
		**Body Weight (kg)**	**Backfat Thickness (mm)**	**Loin Depth (mm)**	**Intramuscular Fat Content (%)**
Duroc (n=205)	Min.	78.93	4.57	27.69	0.38
	Max.	146.97	19.56	64.01	5.07
	Mean	121.04	10.13	49.12	2.07
	SD	11.61	2.55	6.58	0.88
Landrace (n=226)	Min.	91.17	4.83	25.40	0.15
	Max.	152.41	22.10	63.50	4.45
	Mean	123.06	11.59	48.39	1.82
	SD	10.38	2.92	6.66	0.74
Large White (n=220)	Min.	81.65	7.11	26.67	0.30
	Max.	161.93	24.64	61.47	4.03
	Mean	125.75	13.83	46.29	1.98
	SD	12.04	3.24	6.88	0.78
