# Supplementary figures and images for: Use of Host Feeding Behavior and Gut Microbiome Data in Estimating Variance Components and Predicting Growth and Body Composition Traits in Swine
Source: Genes (Basel). 2022 Apr 26;13(5):767. doi: 10.3390/genes13050767 (PMC9140470; doi:10.3390/genes13050767)

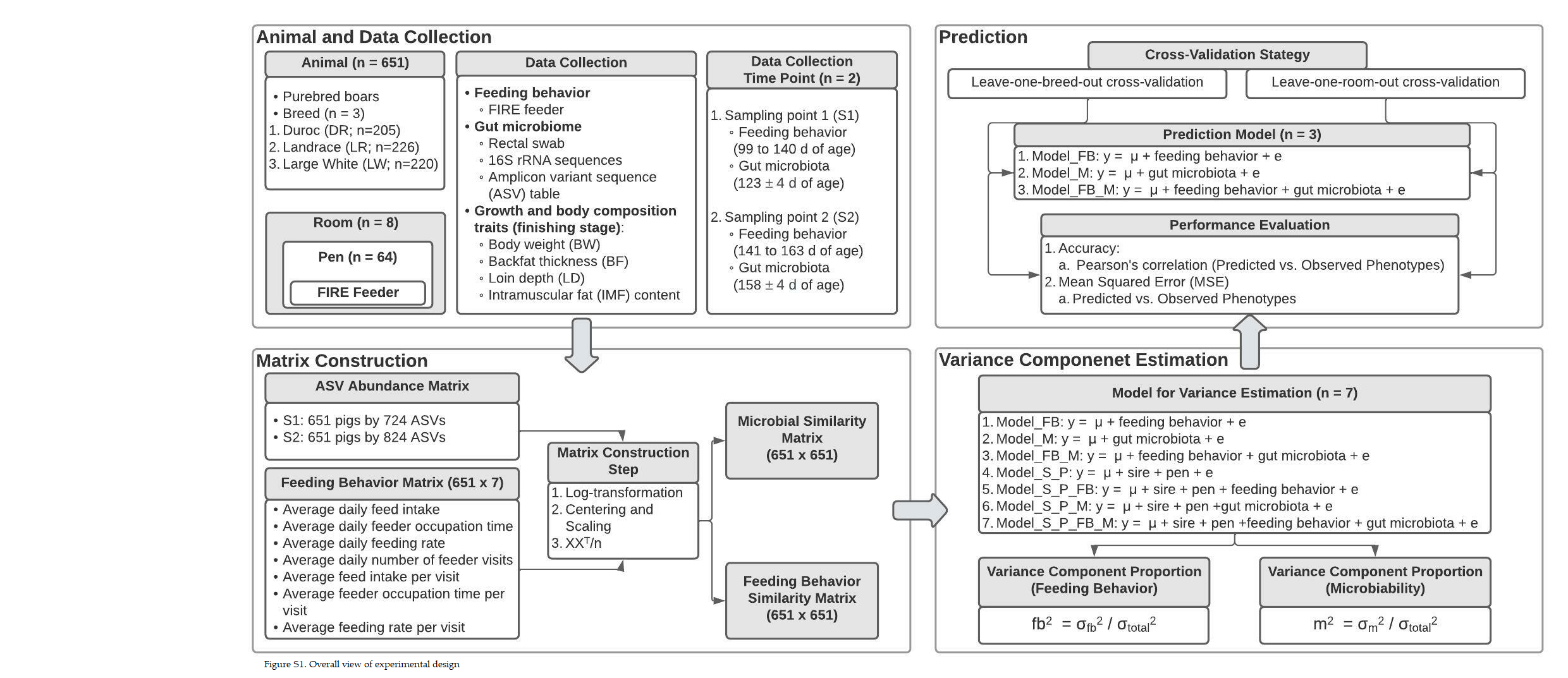

Supplement: Supplementary file 1 [file genes-13-00767-s001.zip › genes-1645048 -supplementary FINAL/FigureS1.png]
